# Targeted protein degradation of PDE4 shortforms by a novel proteolysis targeting chimera

**DOI:** 10.1111/febs.17359

**Published:** 2024-12-13

**Authors:** Yuan Yan Sin, Aoife Giblin, Aleksandra Judina, Punchita Rujirachaivej, Laura G. Corral, Eliza Glennon, Zhi Xian Tai, Tian Feng, Eduardo Torres, Alina Zorn, Julia Gorelik, Elka Kyurkchieva, Pa Thai Yenchitsomanus, Cathy Swindlehurst, Kyle Chan, David Stirling, George S. Baillie

**Affiliations:** ^1^ School of Cardiovascular and Metabolic Health University of Glasgow UK; ^2^ Faculty of Medicine National Heart and Lung Institute, Imperial College London UK; ^3^ Graduate Program in Clinical Pathology, Department of Pathology, Faculty of Medicine Ramathibodi Hospital Mahidol University Bangkok Thailand; ^4^ Katalytic Therapeutics San Diego CA USA; ^5^ Siriraj Center of Research Excellence for Cancer Immunotherapy (SiCORE‐CIT) and Division of Molecular Medicine, Research Department, Faculty of Medicine Siriraj Hospital Mahidol University Bangkok Thailand

**Keywords:** PDE4, phosphodiesterases, PROTAC, protein degradation

## Abstract

Cyclic AMP (cAMP) has a crucial role in many vital cellular processes and there has been much effort expended in the discovery of inhibitors against the enzyme superfamily that degrades this second messenger, namely phosphodiesterases (PDEs). The journey of competitive PDE inhibitors to the clinic has been hampered by side effects profiles that have resulted from a lack of selectivity for subfamilies and individual isoforms because of high conservation of catalytic site sequences and structures. Here we introduce a proteolysis targeting chimera (PROTAC) that can specifically target a small subset of isoforms from the PDE4 family to send the enzyme for degradation at the proteasome by recruiting a ubiquitin E3 ligase into proximity with the PDE. We constructed our PDE4 PROTAC (KTX207) using a previously characterized PDE4 inhibitor, and we show that evolution of the compound into a PROTAC improves selectivity, potency and enables a long‐lasting effect even after the compound is removed from cells after a short treatment duration. Functionally, KTX207 is more effective at increasing cAMP, is 100 times more anti‐inflammatory, and is significantly better at reducing the growth in cancer cell models than the PDE4 inhibitor alone. Our study highlights the advantages of targeted degradation versus active‐site occupancy for PDE4 inhibition and discusses the potential of this novel pharmacological approach to improve the safety profile of PDE4 inhibition in the future.

## Graphical abstract

Here, we report KTX207, a previously undescribed cereblon‐based PDE4 PROTAC that can specifically target PDE4D shortform for degradation. We showed that KTX207 could improve selectivity, potency and enable a long‐lasting effect when used at a sub‐nanomolar concentration. A significant decline in cell growth and TNF‐α inhibition pinpointed the anti‐proliferative and anti‐inflammatory effects of KTX207. Our work highlights the advantages of targeted degradation to establish isoform specificity, which represents an evolution in the development of PDE4 inhibitors.

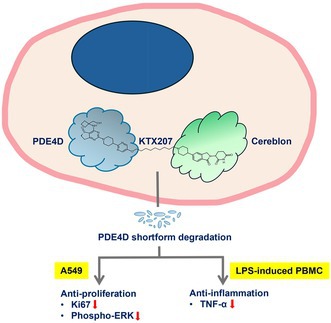

**Abbreviations**
2D: two‐dimensional
3D: three‐dimensional
BTZ: bortezomib
DAPI: 4′,6‐diamidino‐2‐phenylindole
DMSO: dimethylsulfoxide
FRET: Förster resonance energy transfer
FSK: forskolin
GAPDH: glyceraldehyde 3‐phosphate dehydrogenase
IB: immunoblotting
ISO: isoprenaline
LPS: lipopolysaccharide
PARP1: Poly [ADP‐ribose] polymerase 1
PBMCs: peripheral blood mononuclear cells
PDEs: phosphodiesterases
PHA: phytohemagglutinin A
POI: protein of interest
PROTACs: proteolysis targeting chimera
SNIPER: Specific and Nongenetic IAP‐dependent protein erasers
TBST: Tris‐buffered saline with 0.1% Tween 20
TNF‐α: tumor necrosis factor‐α
UCR1: upstream conserved region 1
μM: μmol/L

## Introduction

As cAMP signaling underpins many essential processes in cells, and diminution of the pathway results in a range of disease, much effort has been expended in developing pharmacological inhibitors of the only enzyme superfamily that hydrolyses the cyclic‐nucleotide, namely phosphodiesterases (PDEs) [1]. In particular, the PDE4 family has been a focus as it is the most studied cAMP‐specific PDE family and gives rise to the largest number of isoforms (approximately 25) from the four genes which encode them (PDE4A, 4B, 4C, 4D) [2]. To date, four PDE4 inhibitors have reached the market for a range of conditions with many more under development or in clinical trials [1]. The major barrier to use of PDE4 inhibitors in the clinic has been the lack of selectivity for unique, tissue‐specific PDE4 isoforms, which has resulted in side effects that have limited the therapeutic window [3]. Recently, efforts to discover compounds that can preferentially bind to different PDE4 subfamilies (PDE4A, B, C, D) have used small differences in the structure of the active sites to confer some selectivity [4], though to date no completely subfamily‐specific compounds have been devised. Differences in longform versus shortform PDE4s, which contain different regulatory regions (longforms contain upstream conserved region 1 (UCR1) whereas shortforms do not), have also been exploited to develop longform PDE4‐specific allosteric activators [5] and inhibitors [6], however, no shortform selective compounds have been reported. Overexpression of short PDE4 isoforms is a feature of a diverse set of diseases [7] and they are also upregulated in response to chronic PDE4 inhibition in the treatment of COPD [8].

Targeted protein degradation via proteolysis targeting chimeras (PROTACs) is seen as an alternative to conventional competitive or allosteric inhibition of enzymes. These compounds enter cells and bring together a ternary complex by co‐attracting the protein of interest (POI) and a ubiquitin E3 ligase [9]. The latter ubiquitinates the POI which then gets degraded by the proteasome. The advantages of this approach include concomitant abrogation of enzymatic and nonenzymatic functions of the POI and effective low doses due to the enzymatic action of the PROTAC which sequentially degrades the POI without loss of activity and rapid, long‐lasting action [10]. Particularly relevant to PDE4 inhibition, the design of PROTACs (with variations in warheads (POI ligand), linkers, and E3 ligase recruiters) can be honed to build selectivity [11] among structurally similar isoform targets. Indeed, ‘state‐specific’ PROTACs can be developed with specificity for enzyme targets based on activation level, propensity to oligomerize and cellular location [12], all of which are germane to PDE4.

Here we have developed a PDE4 PROTAC using the PDE4‐specific compound BI 1015550 [13] that has a ninefold selectivity for the PDE4B subfamily over PDE4D as measured in a PDE assay (IC_50_ PDE4B 10 nmoL·L^−1^ vs IC_50_ PDE4D 91 nmoL·L^−1^). As observed in other PROTAC development projects, the efficacy and specificity of the target binder (in this case BI 1015550) can be altered and improved by conversion into a PROTAC [11]. We report that our BI 1015550‐based PROTAC (KTX207) shows a high level of selectivity for PDE4D shortforms, is over a hundred times more potent than the starting point at inhibiting inflammatory markers, and works at an IC_50_ of approximately 10 pm to degrade PDE4D shortforms. Our data indicate that our PROTAC approach could revolutionize the development of novel, potent, long‐lasting and highly selective PDE4 modulators that may result in superior side effect profiles than conventional small molecule inhibitors of the catalytic site.

## Results

### BI‐based KTX207 mediates degradation of PDE4

**Fig. 1:**
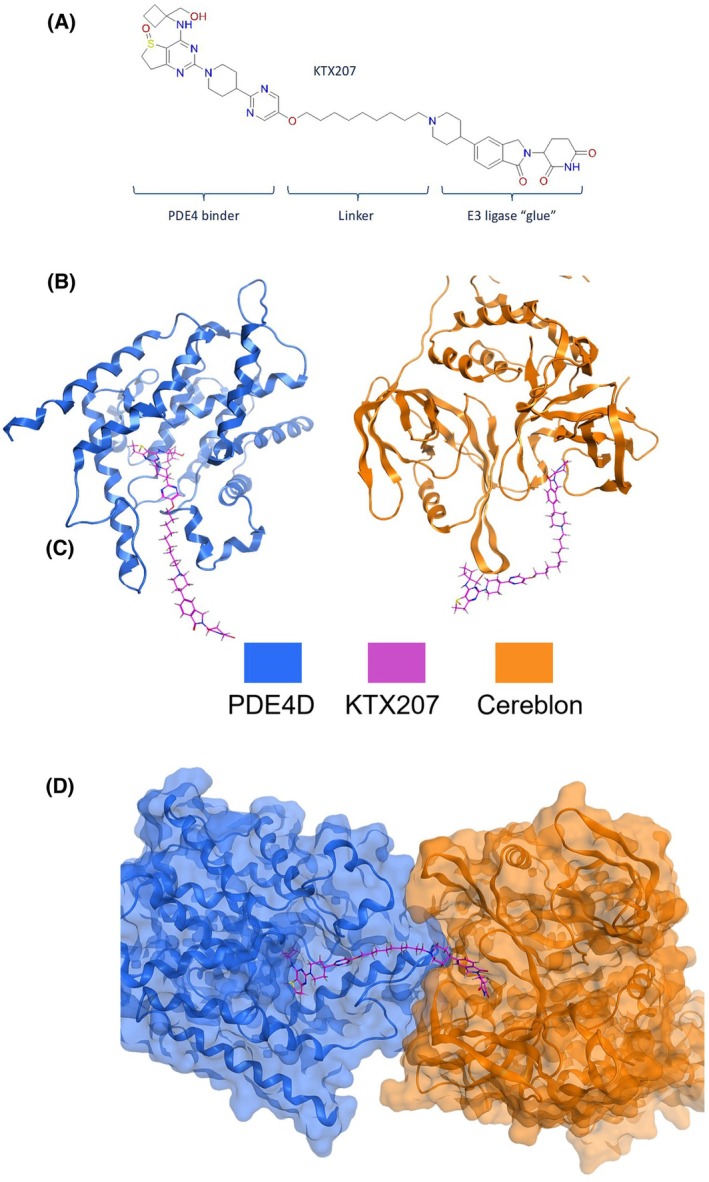
Structure of PDE4‐PROTAC‐Cereblon. (A) The basic structure of KTX207 linking cereblon‐based E3 ligase recruiting moiety to PDE4. (B) Molecular dynamics simulations of KTX207‐mediated complexes using Molecular Operating Environment (MOE) software. Molecular degrader, KTX207‐induced interface contacts with PDE4D (left) and cereblon‐based E3 ligase (right). (C) Ternary complex of PDE4D and cereblon induced by KTX207. Blue cartoons represent PDE4D and orange cartoons represent cereblon E3 ligase. Magenta sticks represent KTX207.

**Fig. 2:**
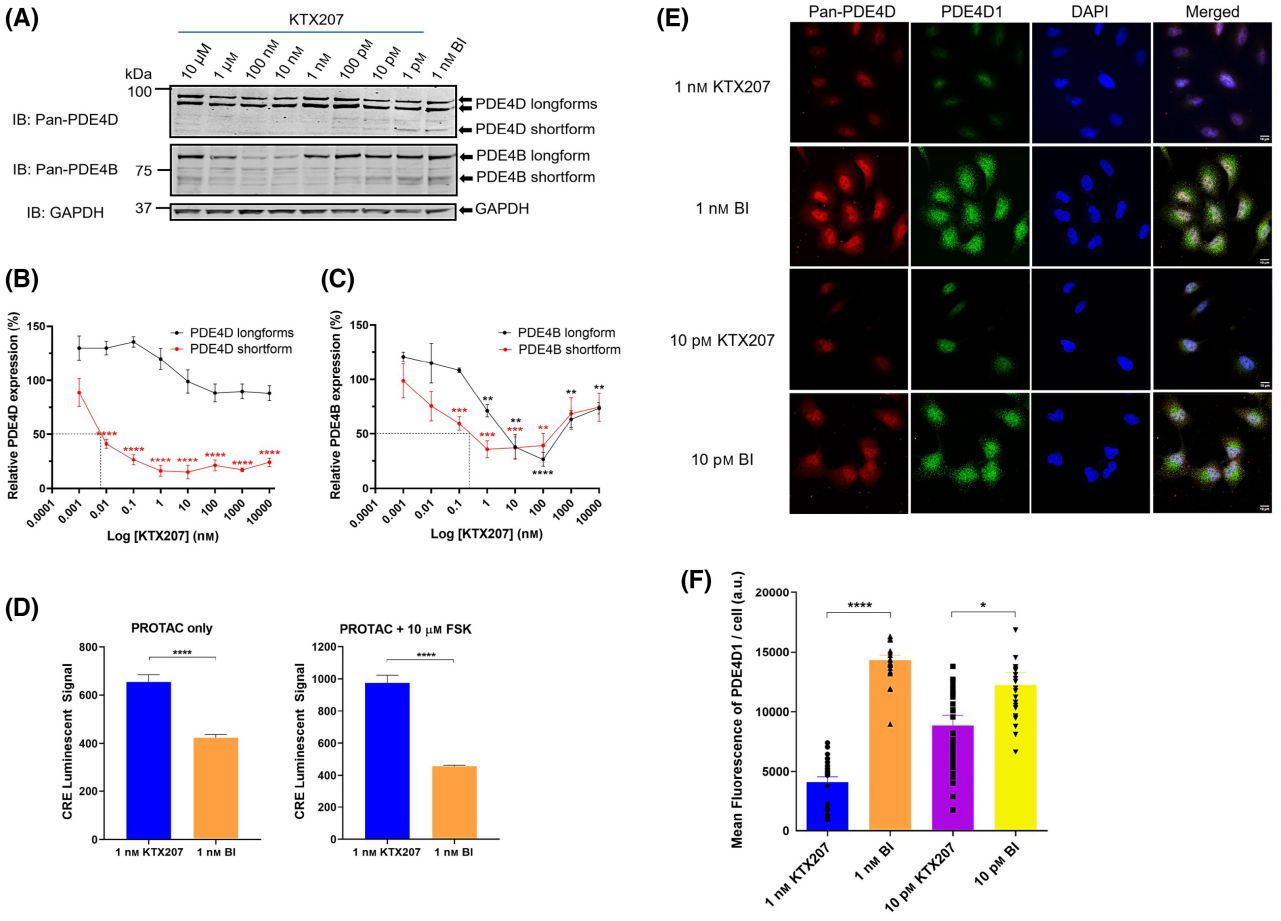
Examination of KTX207‐mediated degradation of PDE4. (A) Immunoblots showing degradation of PDE4D and PDE4B in A549 cells treated with different concentrations of KTX207 for 24 h. GAPDH was used as the loading control. The percentage ratio of (B) PDE4D and (C) PDE4B expression normalized to GAPDH are presented as mean ± SEM (*n* = 4–6). Values of IC_50_ have been estimated. (D) cAMP reporter activation was assessed by signal generated from A549 cells stably expressing cAMP‐responsive luciferase construct. Luciferase activity was measured 24 h after initiation of the reaction by adding 1 nm of compound +100 μm d‐Luciferin with or without 3 h treatment of 10 μm forskolin (*n* = 4). (E) Representative laser scanning confocal micrographs demonstrating the distribution of Pan‐PDE4D (red) and PDE4D1 (green) in A549 cells treated with either 1 nm or 10 pm of compounds for 24 h. Scale bar: 10 μm. (F) Semi‐quantification of the PDE4D1 fluorescent staining compared with the BI control. The results are shown as means ± SEM of several images where at least 4 individual cells were analyzed for each field of view (*n* = 4). All statistical differences were examined by unpaired Student's *t*‐test compared with the BI control. *P < 0.05, **P < 0.01, ***P < 0.001, ****P < 0.0001. IB, immunoblotting with the indicated antibody.

KTX207 is a PDE4 degrader that uses a warhead based around the PDE4 inhibitor BI 1015550 [13, 14] which has ninefold preference for PDE4B (10 nm IC_50_) over PDE4D (91 nm IC_50_). The basic structure of KTX207 is depicted in Fig. 1A. Molecular modeling of both bi‐molecular complexes between 1. The PDE4 catalytic unit and BI 1015550‐linker (Fig. 1B, left panel) and 2. Isoindolinone core cereblon and the alkyl‐chain‐linker (Fig. 1B, right panel) show the possible docking position of the enzyme recruiting moieties and exit trajectories for the linker. The ternary complex model (Fig. 1C) illustrates that KTX207 enables proximity of the E3 ligase and the PDE that is essential for the ubiquitination and proteasome‐mediated degradation of the PDE4. We selected A549, a human lung adenocarcinoma cell line to test our PDE4 degraders as previous research had reported protein expression of long and short isoforms from all 4 subfamilies [15, 16]. Western blotting confirmed that the cells expressed PDE4B/PDE4D long and short forms (Fig. 2). In dose–response studies, KTX207 degraded PDE4D (Fig. 2A, upper panel and Fig. 2B) and PDE4B shortforms (Fig. 2A, lower panel and Fig. 2C) with an IC_50_ of 10 pm and 400 pm, respectively. KTX207 was highly selective for PDE4 shortforms over longforms as PDE4D longforms were not significantly degraded at any of the concentrations tested, whereas PDE4B longforms were significantly degraded to <50% of control levels at 10 nm (Fig. 2B,C). Interestingly, the PDE4B longform degradation dose–response curve (Fig. 2C) exhibited a classical hook effect [17] showing activity in a concentration range that reflected assembly of ternary complexes as opposed to concentrations that promote only a binary complex of the PROTAC molecule to either the PDE4 or the E3 ligase. The observed degradation of PDE4 resulted in significant increases in cAMP under basal conditions (Fig. 2D, left panel) and following adenylate cyclase activation by forskolin (Fig. 2D, right panel) when comparing the PROTAC (KTX207) to the BI 1015550 warhead alone. Confirmation of intracellular PDE4 degradation was confirmed using confocal microscopy and antibodies against pan‐subfamily PDE4D and the specific short isoform, PDE4D1 (Fig. 2E). Treatment with two different concentrations (1 nm and 10 pm) of KTX207 (but not BI 1015550 alone (Ctrl)) resulted in a significant reduction in fluorescence intensity for both pan PDE4D and PDE4D1 underlining the effectiveness of the PDE4 degrader and its ability to cross cell membranes (Fig. 2F).

### KTX207 degrades PDE4D shortform with high selectivity at sub‐nanomolar concentration via the ubiquitin‐proteasomal system

**Fig. 3:**
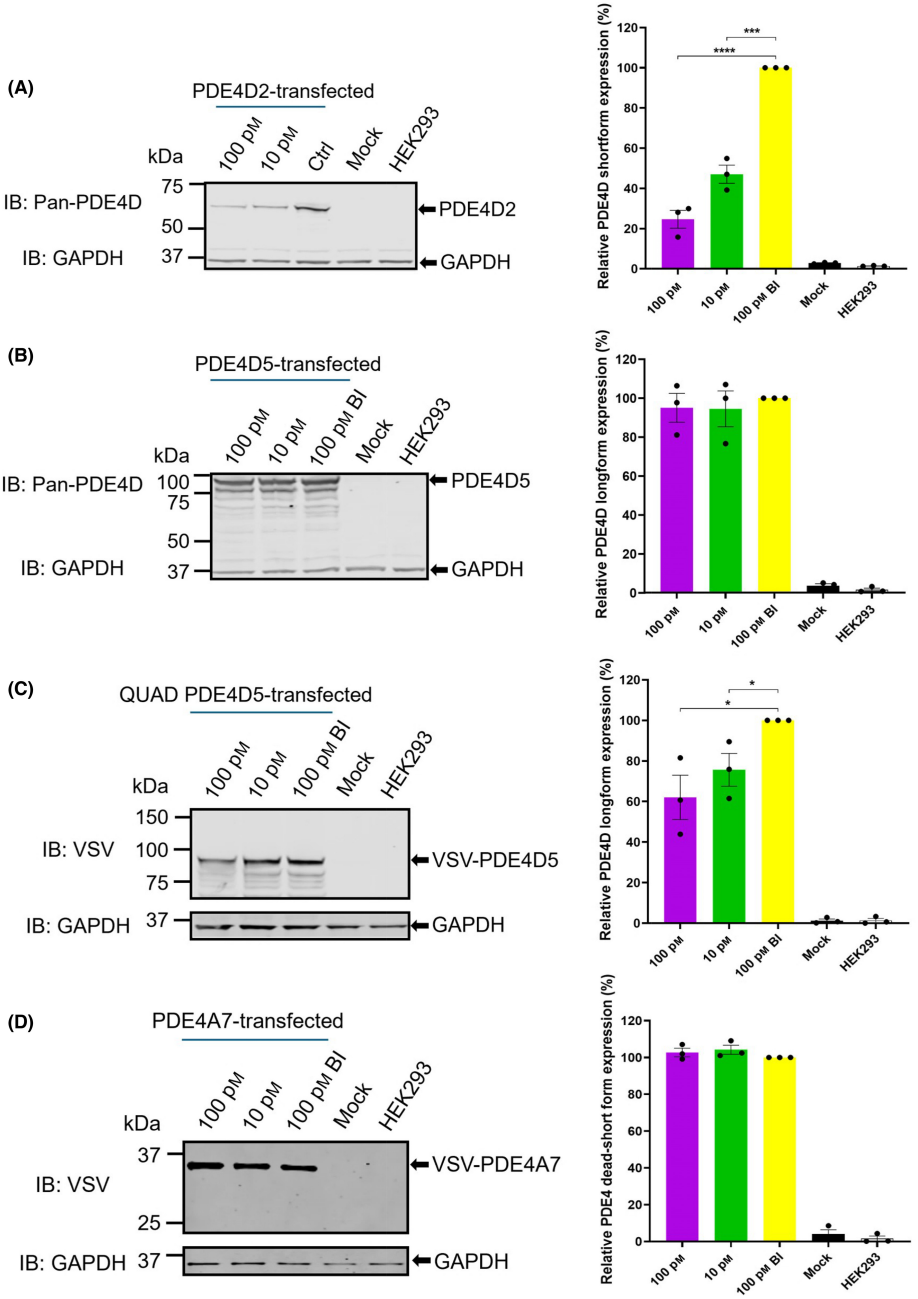
KTX207 degrades PDE4D shortform with high selectivity. Representative immunoblot showing (A) degradation of PDE4D shortform in HEK293 cells overexpressing PDE4D2, (B) degradation of PDE4D longform in HEK293 cells overexpressing PDE4D5, (C) degradation of PDE4D longform in HEK293 cells overexpressing Quad‐PDE4D5 (K48R:K53R:K78R:K140R) mutant, (D) degradation of PDE4 dead‐short form in HEK293 cells overexpressing PDE4A7 following 24 h treatment with indicated concentrations of KTX207. The percentage ratio of PDE4D expression normalized to GAPDH are presented as mean ± SEM (*n* = 3). All statistical differences were examined by unpaired Student's *t*‐test compared with the BI control. *P < 0.05, **P < 0.01, ***P < 0.001, ****P < 0.0001. IB, immunoblotting with the indicated antibody.

Confirmation of the PDE4 shortform selectivity of KTX207 was achieved using a transiently transfected PDE4D shortform PDE4D2 (Fig. 3A) and PDE4D longform PDE4D5 (Fig. 3B). Significant degradation of PDE4D2 but not PDE4D5 was observed following treatment with 100 pm KTX207. To determine if dimerization of PDE4 longforms is a factor in the mechanism that precludes them from degradation by KTX207, a mutant that has previously been shown to attenuate dimerization (QUAD PDE4D5) [18] was transfected into HEK293 cells (Fig. 3C). Interestingly, both 10 pm and 100 pm KTX207 treatments produced a significant degradation of the QUAD PDE4D5 mutant suggesting that monomeric forms of PDE4 may allow a more efficient formation of the tetrameric complex (PDE4‐PROTAC‐Cereblon) that promotes PDE4 degradation. As the PROTAC needs to bind with the catalytic unit of the PDE4 to promote proteolysis, it was unsurprising that the ‘dead’ shortform PDE4A7 was not degraded by KTX207 (Fig. 3D) as it has a truncated catalytic domain [19].

**Fig. 4:**
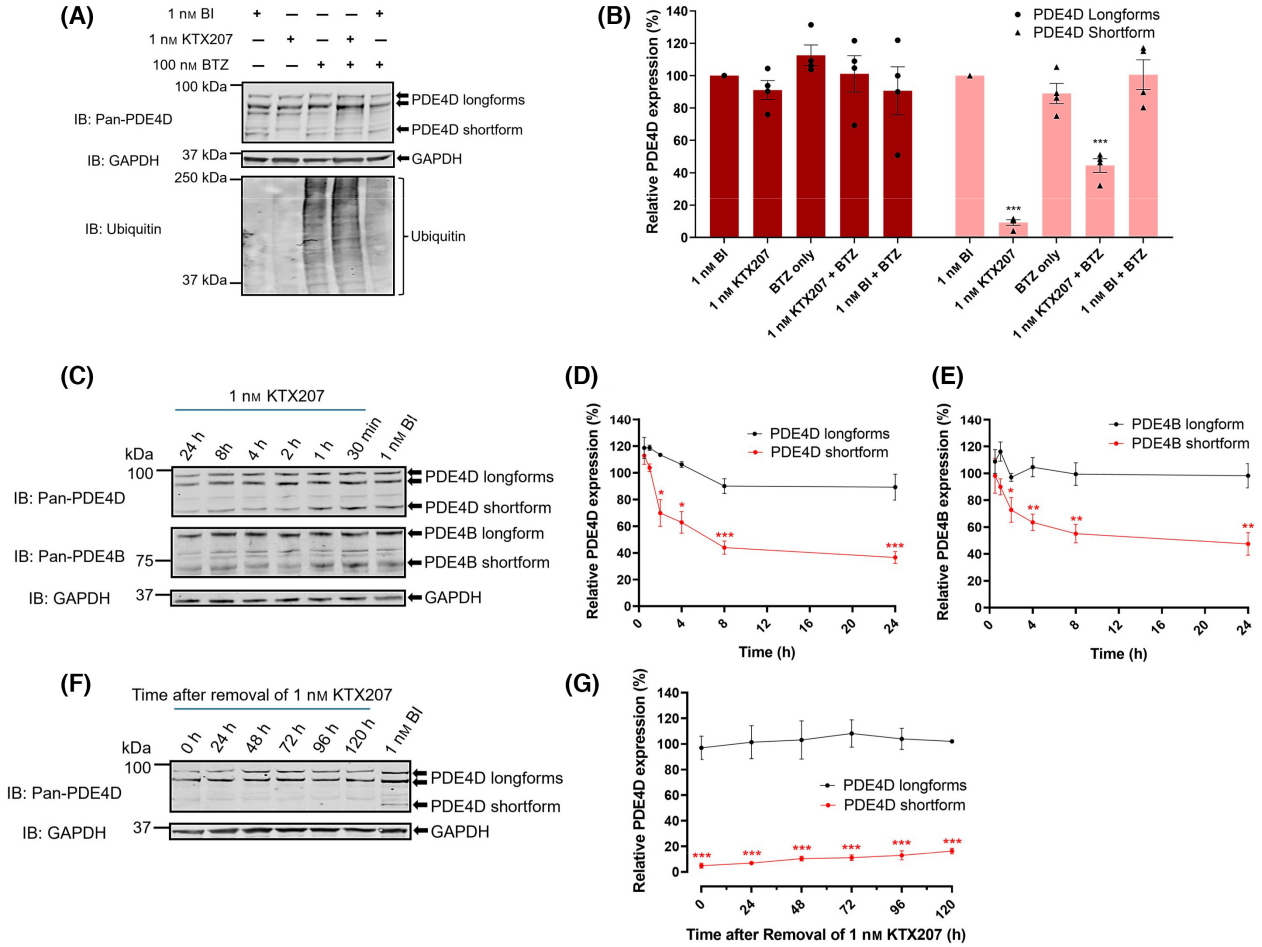
Efficacy of KTX207‐mediated degradation of PDE4D shortform. (A) Proteasome‐dependent degradation of PDE4D shortform by KTX207. A549 cells were treated with compounds with or without 100 nm of proteasome inhibitor, bortezomib (BTZ) which caused accumulation of poly‐ubiquitinated proteins in A549 cells. GAPDH was used as the loading control. (B) PDE4D protein levels normalized to GAPDH (*n* = 4). (C) Onset of action of KTX207‐mediated degradation of PDE4. Immunoblot analysis of (D) PDE4D and (E) PDE4B expression after treatment with 1 nm of KTX207 at indicated timepoints. PDE4D protein levels normalized to GAPDH (*n* = 3). (F) Long‐lasting effect of KTX207. A549 cells were treated with 1 nm of KTX207 for 24 h. Then, the medium was removed and fresh medium without compounds was added. Protein lysates were collected after indicated time and analyzed by western blotting. (G) The percentage ratio of PDE4D expression normalized to GAPDH are presented as mean ± SEM (*n* = 4). All statistical differences were examined by unpaired Student's *t*‐test compared with the BI control. *P < 0.05, **P < 0.01, ***P < 0.001. IB, immunoblotting with the indicated antibody.

Next, to prove that degradation of PDE4D shortforms was mediated via the ubiquitin‐proteasome system we cotreated A549 cells with a proteasome inhibitor, bortezomib (BTZ) and KTX207 to illustrate that the degradation of PDE4D shortforms could be significantly rescued (Fig. 4A,B). Increased ubiquitination of the entire proteome was evident in bortezomib treated cells (Fig. 4A, lower panel) and the level of degradation of PDE4D shortforms promoted by KTX207 was significantly attenuated following cotreatment (Fig. 4A upper panel, lane 2 vs lane 4 and Fig. 4B).

As PROTAC compounds cross the cell membrane and recruit PDE4 and the appropriate E3 ligase, we wished to determine the time to action of KTX207. Significant degradation of both PDE4B and PDE4D shortforms was recorded only 2 h after treatment and this continued up until 24 h (Fig. 4C). PDE4B and PDE4D longforms were not degraded over this time at this concentration of KTX207 (1 nm) (Fig. 4D,E). Protein degraders sequentially degrade their targets once they reach the cytoplasm and considering this, we wanted to determine the longevity of action of a single dose (1 nm) that was removed from the cells after a 24‐h treatment (Fig. 4F). Surprisingly, there was very little recurrence of the PDE4D shortform even 120 h from washout (Fig. 4G), signifying that once inside the cells, the PDE4 shortform degraders were still highly active in preventing detectable levels of the enzyme protein.

### KTX207 is more effective at increasing cAMP levels than conventional inhibitor

**Fig. 5:**
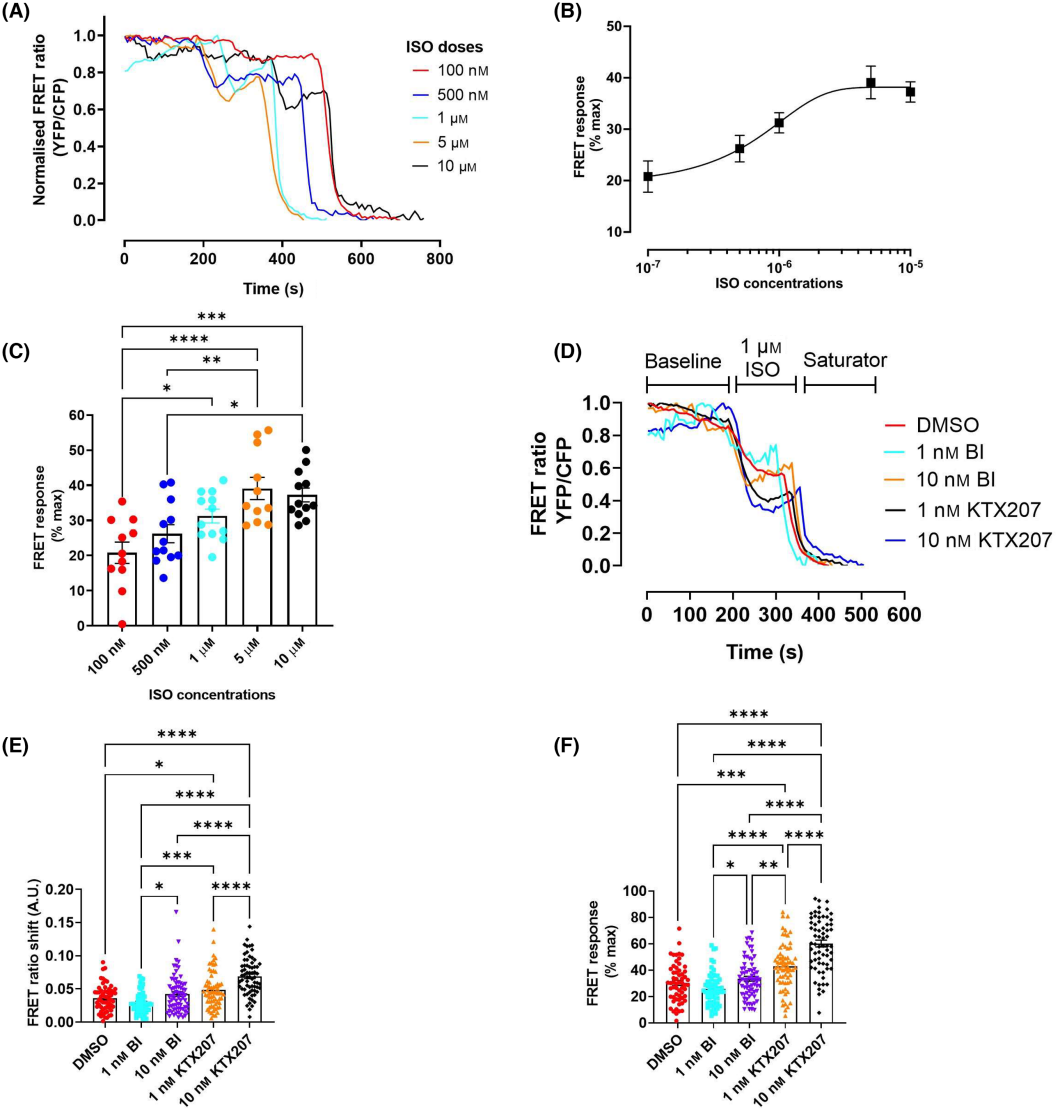
Cytosolic FRET response of A549 cells expressing Epac1‐camps FRET sensor. (A) Representative FRET curve, (B) Log(agonist) vs. response line chart, and (C) bar chart normalized to isoprenaline (0.1, 0.5, 1, 5, and 10 μm) stimulation followed by saturator (10 μm forskolin and 100 μm IBMX). Data expressed as mean ± SEM; *n* = 11 cells; *P < 0.01, **P < 0.01, ***P < 0.001, ****P < 0.0001 based on one‐way ANOVA with Tukey's multiple comparisons test. (D) Representative FRET curve, (E) bar chart of FRET ratio shift, and (F) bar chart of normalized FRET response to isoprenaline stimulation of 1 nm BI (cyan), 10 nm BI (violet), 1 nm KTX207 (green), 10 nm KTX207 (purple), and DMSO‐treated control (red). Data expressed as mean ± SEM; *n* = 59 cells from three independent experiments; *P < 0.05, **P < 0.01, ***P < 0.001, ****P < 0.0001 based on one‐way ANOVA with Holm‐Šídák's multiple comparisons test.

To determine whether the profound degradation of shortform PDE4s triggered by KTX207 resulted in altered cAMP dynamics, we utilized a genetically encoded cAMP reporter based on the structure of EPAC [20]. To sample physiologically relevant concentrations of cAMP, we decided to evaluate cAMP production following stimulation of the beta‐adrenergic system which already had been shown to exist in A549 cells [21]. Initial experiments using isoprenaline (traces depicted in Fig. 5A) showed a dose‐dependent increase in cAMP that peaked at 5 μm (Fig. 5B,C). Using 1 μM isoprenaline in all other experiments, we were able to show that the BI 1015550 compound was ineffective at 1 nm or 10 nm in enhancing cAMP following isoprenaline treatment (Fig. 5D). KTX207 on the other hand, significantly enhanced the cAMP response at 1 nm and 10 nm when compared with DMSO control or the BI 1015550 compound demonstrating the superior PDE4 abrogation elicited by the PDE4 degrader (Fig. 5D). The conclusion was the same if we compared degrader to warhead alone using absolute FRET shift data (Fig. 5E) or comparison to the saturated response (Fig. 5F). The BI 1015550 targeted PROTAC was significantly better at raising cAMP than BI 1015550 alone.

### KTX207 has a higher anti‐inflammatory potency than conventional inhibitor

**Fig. 6:**
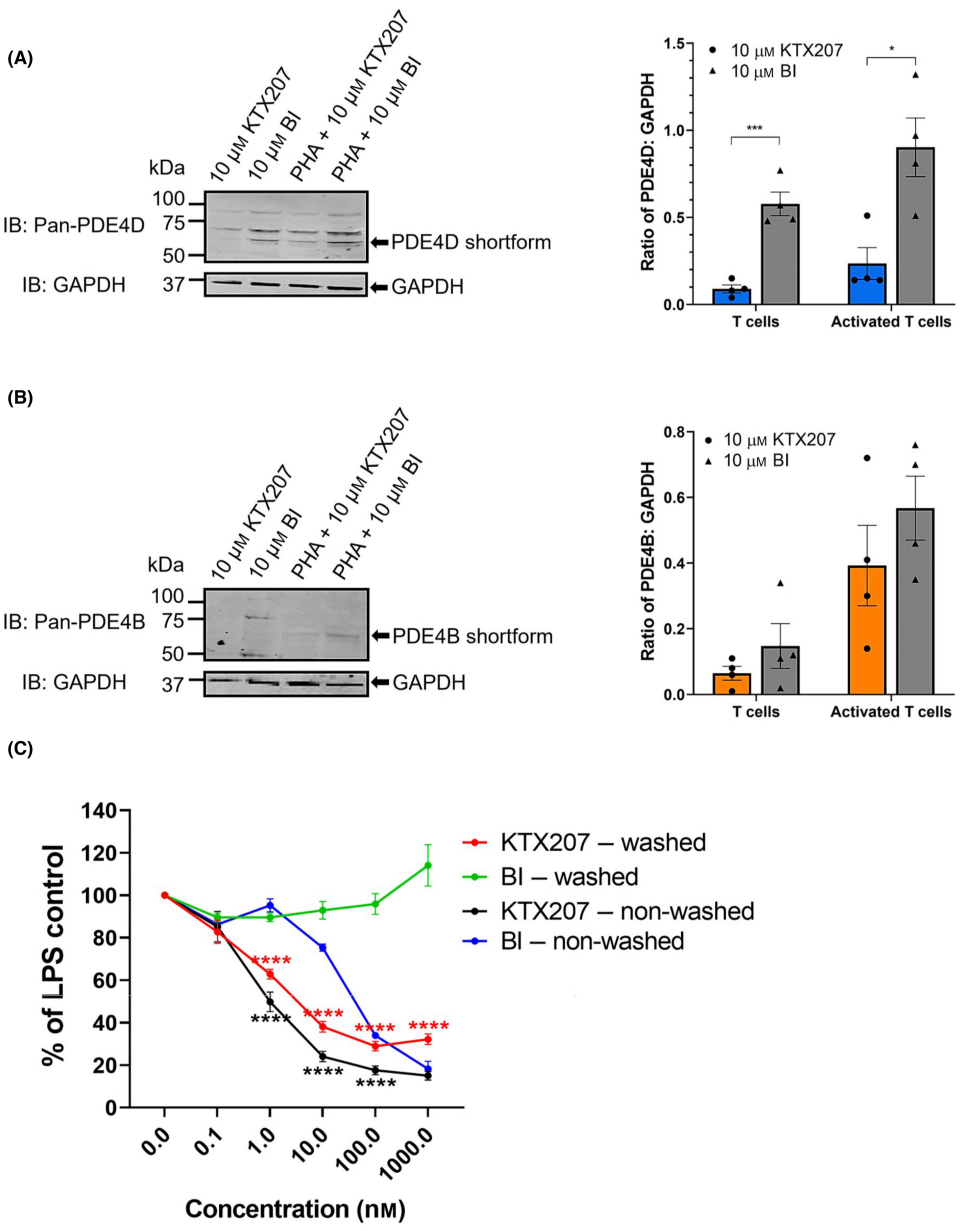
Functional readout for KTX207‐mediated PDE4 degradation. Representative immunoblots showing (A) PDE4D and (B) PDE4B expressions in T cells and PHA‐activated T cells (left panel). Quantification of PDE4 expression normalized to GAPDH and present as mean values ± SEM (*n* = 4) (right panel). (C) LPS‐induced production of TNF‐α by PBMC with or without removal of KTX207. Data are presented as mean ± SEM (*n* = 3). All statistical differences were examined by unpaired Student's *t*‐test compared with the BI control. *P < 0.05, **P < 0.01, ***P < 0.001, ****P < 0.0001. IB, immunoblotting with the indicated antibody.

Next to find a functional readout for PDE4 degradation by KTX207, we looked at release of the pro‐inflammatory cytokine, tumor necrosis factor‐a (TNF‐a). Previously, BI 1015550 had been shown to inhibit TNF‐a release in human peripheral blood mononuclear cells (PBMCs) stimulated with phytohemagglutinin B (PHB) (BI 1015550 IC_50_: 9 nm) and LPS (BI 1015550 IC_50_: 35 nm) [13]. Here we stimulated human PBMCs with phytohemagglutinin A (PHA) to determine if we could visualize the reported increase in PDE4 shortforms previously reported [22, 23] as part of the pro‐inflammatory response. In agreement with the earlier studies, expression of shortforms from the PDE4D (Fig. 6A) and PDE4B (Fig. 6B) subfamilies was low but increased upon PHA treatment. The increases elicited by PHA in PDE4D shortforms were significantly attenuated by KTX207 (Fig. 6A). When we evaluated the release of TNF‐a from PBMCs stimulated with LPS that had been either treated with BI 1015550 or KTX207, we discovered that the PROTAC was 100 times more potent than the conventional inhibitor control in inhibiting TNF‐a release (IC_50_ 1 nm vs IC_50_ 100 nm) (Fig. 6C). Additionally, when both BI 1015550 or KTX207 were washed away from the human PBMCs, only the PROTAC continued to inhibit TNF‐a release due to the catalytic mechanism of action (Fig. 6C). These data validate the notion of a potent long‐lasting PROTAC effect as seen in the previous washout experiment (Fig. 4F,G).

### KTX207 can suppress cancer cell growth in 2D and 3D cultures of A549 cells

**Fig. 7:**
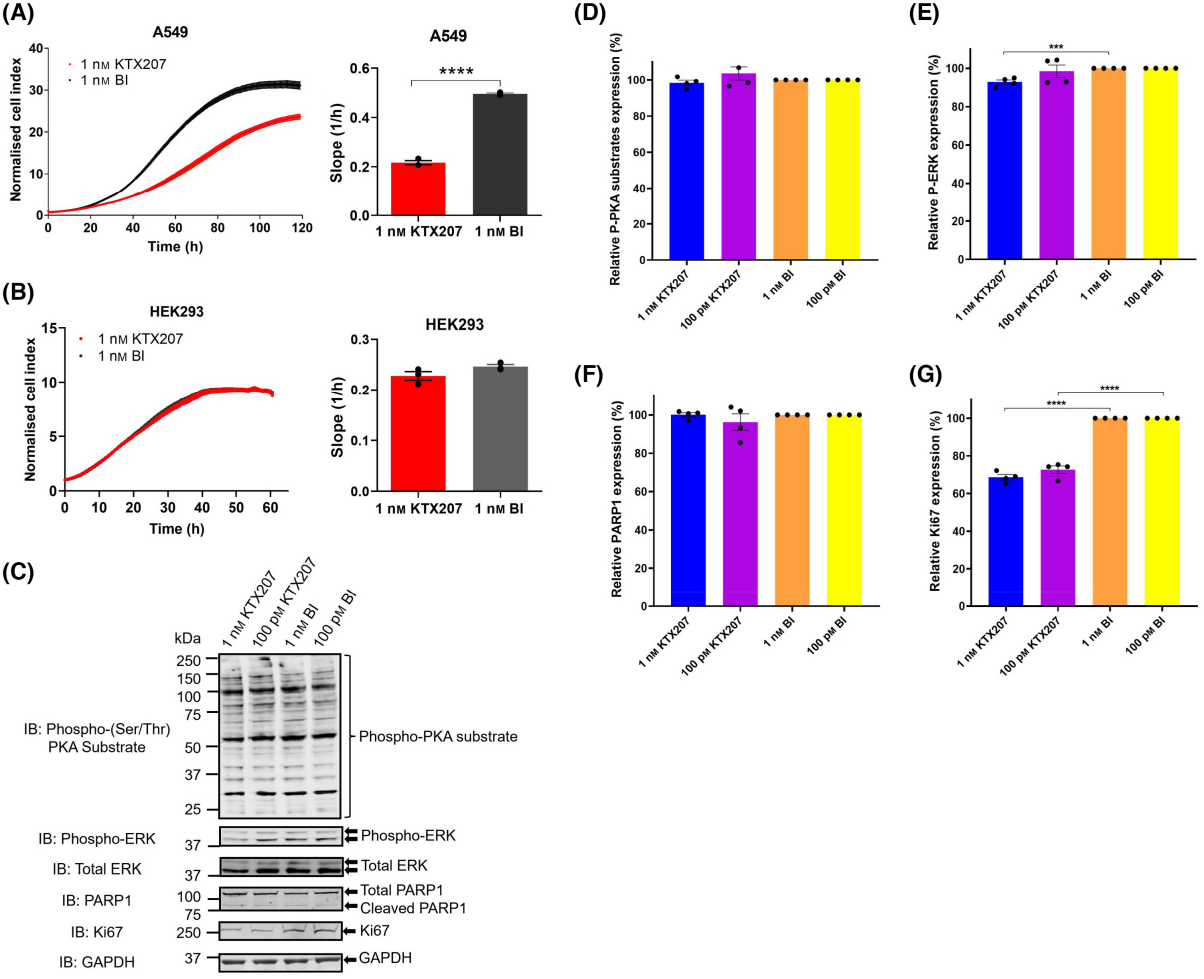
KTX207 suppresses cancer cell growth in A549 cells. (A) Real‐time cell proliferation of A549 cells monitoring by xCELLigence system (left panel). Continuous impedance measurement displayed as normalized cell index. Data were normalized to the time of compound addition (T = 0 h). The rate of A549 cell proliferation was determined by measuring the slope of the line between the 25‐ and 65‐h interval (right panel). (B) xCELLigence real‐time cell analysis of HEK293 cells (left panel). The rate of HEK293 cell proliferation was determined by measuring the slope of the line between the 10‐ and 35‐h interval (right panel). (C) Representative immunoblots showing phospho‐PKA substrate, phospho‐ERK, total ERK, PARP1, and Ki67 in A549 cells after 24 h of treatment as indicated. GAPDH was the loading control. The percentage ratio of (D) phospho‐PKA substrate, (E) phospho‐ERK, (F) PARP1, and (G) Ki67 expressions normalized to GAPDH and are presented as mean ± SEM (*n* = 4). All statistical differences were examined by unpaired Student's *t*‐test compared with the BI control. ***P < 0.001, ****P < 0.0001. IB, immunoblotting with the indicated antibody.

**Fig. 8:**
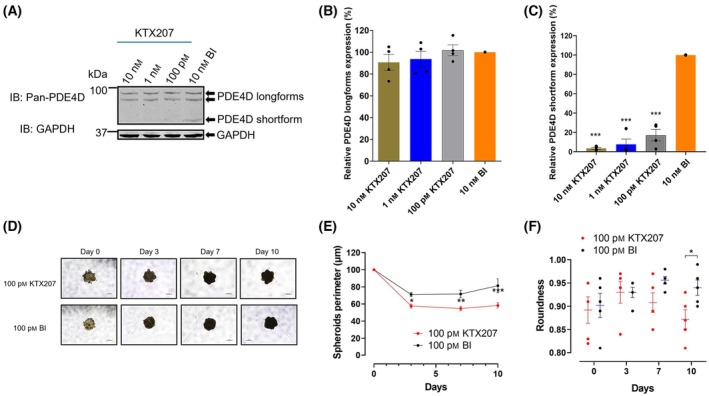
Growth kinetics of A549‐derived tumor spheroids. (A) Immunoblot showing degradation of PDE4D in A549 spheroids treated with different concentrations of KTX207 for 24 h. GAPDH was used as the loading control. The percentage ratio of (B) PDE4D longforms and (C) PDE4D shortform expressions normalized to GAPDH are presented as mean ± SEM (*n* = 4). (D) Representative brightfield images showing morphology of A549 cell line cultured as 3D spheroids in ultra‐low attachment 96‐well plates. Scale bar: 200 μm. The images were captured by a phase‐contrast microscopy at day 0, 3, 7, and 10 in culture treated with 100 pm of compounds (*n* = 5). (E) Time‐course monitoring of spheroid growth expressed in perimeter (microns) and (F) the roundness of spheroids evaluated over a period of 10 days (*n* = 5). All statistical differences were examined by unpaired Student's *t*‐test compared with the BI control. *P < 0.05, **P < 0.01, ***P < 0.001, ****P < 0.0001. IB, immunoblotting with the indicated antibody.

In searching for another indication for KTX207, we utilized the fact that certain cancers have homozygous microdeletions in the human PDE4D gene locus that do not affect presence of the mRNA nor inactivate the protein but partially remove the UCR1 domain enhancing the likelihood of a ‘short’ type PDE4D [15]. As short PDE4D isoforms are unaffected by autoinhibition and are more active, therefore promoting proliferation of tumor cells, we looked at the two‐dimensional (Fig. 7) and three‐dimensional growth of A549 cells (Fig. 8) which have been shown to be sensitive to PDE4D silencing [15] or inhibition [16]. Using xCELLigence real‐time cell analysis, we saw that reduction of PDE4D and PDE4B shortforms using 1 nm KTX207 (Fig. 2) resulted in a significant decrease in A549 growth when grown in two dimensions (Fig. 7A). A downregulation of phospho‐ERK and Ki67 were seen in the KTX207‐treated cells (Fig. 7C–G). No effect was seen on the noncancerous kidney cell line HEK293 (Fig. 7B). To better mimic the situation *in vivo*, we cultured A549 spheroids and demonstrated that KTX207 was effective in penetrating the three‐dimensional cultures to promote significant degradation of PDE4D shortform (Fig. 8A,C) but not PDE4 longforms (Fig. 8A,B). Treatment with KTX207 also significantly reduced the diameter of A549 spheroids (Fig. 8D,E) and affected the roundness of them after 10 days (Fig. 8F) signifying that our PDE4 shortform degrader slowed growth and promoted deformation of the cancer cell mass.

## Discussion

As PDE4 enzymes are encoded by four genes that encode approximately 25 different isoforms which differentially locate in cells and tissue, their function has been intricately linked to their incorporation into cAMP nano‐domains with cells [24]. Recent developments in cAMP reporter technology and mathematical modeling of cAMP dynamics [25] have allowed appreciation of the coordinating role PDE4s have in underpinning apt spatial and temporal cAMP signaling in cells and organs [26] to produce appropriate physiological outcomes in response to the activation specific Gs‐coupled receptors [27]. Designing PDE4‐specific inhibitors that can target subfamilies or individual isoforms of the enzyme family has been difficult due to extremely high similarity in the structures between the active sites of subfamilies [4]. Different approaches that target PDE4 enzyme location in cAMP signaling complexes have utilized tools that displace precisely located PDE4s to decipher different functionalities even between spatially distinct ‘pools’ of the same isoform [28, 29]; however, the peptidic nature of these has precluded these agents from further development as therapeutics.

Targeted protein degradation is a relatively new concept in pharmacology that allows specific target selection of a protein of interest combined with rapid destruction of that target by the 26S proteosome [30]. This process relies on molecular glues or PROTACs, which induce non‐native interactions with ubiquitin E3 ligases, condemning the POI to proteolysis following its covalent modification by ubiquitin. Recently, this strategy has been applied to develop inhibitors for PDE6 and PDE4. In the former case, the pde6d gene encodes a subunit that is known for its ability as a prenyl‐binding protein [31]. The protein product, PrBP/d or PDE6d, is an important shuttling factor for KRAS, which enables the kinase to be positioned correctly on the plasma membrane where it acts as a tightly controlled signal transduction switch. KRAS activates effector proteins following their translocation to membrane and many diseases including a range of cancers result from gain‐of‐function mutations that persistently activate the kinase [32]. Protein–protein interaction disruptor molecules that displace PDE6d from KRAS by binding to the prenyl‐binding pocket of the PDE proved to be effective in inhibiting oncogenic KRAS signaling and showed some efficacy in models of pancreatic cancer at micromolar concentrations [33]. Excitingly, use of the PDE6d‐KRAS disruptor compounds as warheads for the development of PROTACS enabled rapid and specific PDE6d degradation greatly improved the potency of the compounds, enhanced their ability to inhibit ERK map kinase signaling, and augmented their anti‐proliferative activity against colorectal cancer *in vitro* and *in vivo* [34].

In the case of PDE4, ligands that recruit inhibitor of the apoptosis protein (IAP) E3 ligases were conjugated to an active‐site directed PDE4 inhibitor (piclamilast analogue developed for dry eye syndrome [35]) via a linker in order to construct a PDE4 SNIPER (specific and nongenetic IAP‐dependent protein erasers) [36]. Significant degradation of endogenously expressed PDE4A longforms was observed after 6 h treatment with the PDE4 SNIPER in HT1080 cells (human epithelial cells from fibrosarcoma patient), and this could be rescued by pretreatment with a proteasome inhibitor validating the mechanism of action. No information with respect to degradation of the other PDE4 subfamilies was given. Additionally, although extensive development of IAP‐based SNIPER PROTACS against a range of proteins implicated in cancer, immune disease, and neurodegenerative diseases has followed since their initial discovery [37], the PDE4 SNIPER has not been taken forward, probably due to lack of potency, ‘flat’ dose–response curve and inefficient proteolysis of the PDE4 target which left over 40% of the starting protein intact at high nanomolar concentrations [36]. This is in stark contrast to our PDE4 PROTAC which drives a more complete proteolysis of PDE4D shortforms with a pronounced dose–response that demonstrates an IC_50_ for degradation of approximately 9 pm (Fig. 2). There are also parallels with the PDE6d PROTAC development as our evolution of BI 1015550 into a selective degrader has enhanced its ability to inhibit TNF‐a release in LPS stimulated PBMCs from an IC_50_ of 100 nm for the active‐site directed inhibitor (35 nm reported in [13]) versus approximately 1 nm for the BI 1015550 targeted PROTAC (Fig. 6C). With respect to PDE4D inhibition, the BI 1015550 compound inhibited PDE4D activity with an IC_50_ of 91 nm [13] whereas we could affect a 50% reduction in PDE4D shortform protein with only 9 pm KTX207. The differential effects of PDE4 inhibition between BI 1015550 and KTX207 were also evident when looking at cAMP dynamics as the PROTAC resulted in a significantly greater cAMP response following beta‐adrenergic stimulation or forskolin treatment compared to BI 1015550 (Figs 1E, 4). These noteworthy enhancements are likely due to the catalytic nature of the degrader, which acts sub‐stoichiometrically, allowing one PROTAC molecule to induce multiple rounds of protein degradation in series. Due to this event‐driven mode of action, degraders tend to work at lower concentrations than conventional pharmaceuticals and are much less likely to trigger off‐target toxicity because there is less reliance on high‐target occupancy [38]. Hence, the concept that conventional PDE4 inhibitors can be markedly improved by conversion into PROTACS is proven in the case for BI 1015550 and it will be exciting to ascertain in future work if this holds true for other characterized PDE4 inhibitors.

With respect to selectivity, BI 1015550 is marketed as a selective ‘PDE4B inhibitor’ even although it only has a ninefold selectivity for PDE4B over PDE4D: 10 nm vs 91 nm IC_50_ [13]. KTX207, in comparison, is specific for PDE4D shortforms at 10 pM and this represents the first demonstration of a concentration‐dependent shortform PDE4 degrader and a PDE4D‐specific compound (Fig. 2). PDE4D longform selective, allosteric compounds are under investigation for a range of brain disorders [39, 40] and their selectivity arises from a single amino acid difference (PDE4D vs PDE4A, 4B, 4C) in the UCR2 region [6]. As shortforms do not contain this region [7], the GEBR compounds are specific for longforms but only highly selective (approximately 100‐fold) for the PDE4D sequence over other subfamilies. The mechanism behind the subfamily specificity of KTX207 is currently unknown; however, there are several possibilities that have come to light during the discovery and development of PROTACS for other proteins [12]. It is known that different conformations of the same protein (e.g., BRAF [41]) can enhance PROTAC action presumably by presenting a more favorable orientation for lysine ubiquitin by the E3 ligase recruited. The oligomerization state of the POI is also a consideration, and degraders have been developed for Huntington's disease that target aggregated mutant huntingtin (mHTT) but not the wild‐type HTT [42]. This was possible by firstly identifying warheads that selectively bind mHTT and building the PROTACS around them. Dimerization interfaces can also be used to target PROTACS and compounds that send monomeric survivin for destruction by the proteasome have been developed for use in prostate cancer [43]. This is relevant to the structure of PDE4s, where longforms are known to dimerize, whereas shortforms remain monomeric [18, 44] and it could be that KTX207 selects only monomeric PDE4s because of their conformation or that the dimer occludes binding of the large PROTAC structure. This concept is supported by our work using the monomeric longform PDE4D5 mutant (Fig. 2C), but further work based on the selectivity of KTX207 is required.

As we have fully characterized PDE4 shortform degradation in A549 cells, we chose this cell line for functional studies. This cell line was included in a previous study that charted homozygous PDE4D microdeletions that partially or fully removed UCR1/UCR2 regions to elevate PDE4D enzyme activity and promote survival of cancer cells [15]. In light of this, use of our PDE4D shortform targeting degrader, KTX207, appeared to be an apt approach as use of the PDE4D selective inhibitor 26b [45], triggered cell death and growth retardation in cells carrying the microdeletions [15]. Additionally, a different PDE4 inhibitor (NCS 613) has also been shown to have anti‐inflammatory and anti‐proliferative actions in A549 cells [16], so the rationale for using our PDE4 degrader cell line was clear. Treatment with KTX207 degraded PDE4 shortforms in 2D cultures (Fig. 1B) and in 3D spheroids (Fig. 7A,C). In both models, KTX207 resulted in a significant retardation of cell proliferation (Figs 6A, 7D,E) and in the case of the 3D model reduced roundness after 10 days (Fig. 7F). In addition, there was a reduced expression in phospho‐ERK and Ki67 (Fig. 6C,E,G) which are normally expressed in proliferating cells during active phases of the cell cycle. These growth reductions agreed with the previous papers but did not reach the extent of growth inhibition seen with conventional PDE4 inhibitors, although the other studies only realized significant effects at micromolar concentrations compared to one nanomolar concentration of KTX207 in our work. It is also possible that the inhibition of the PDE4 longforms expressed in A549 had a bearing on the success of Pan‐PDE4 inhibition as many PDE4 longforms have been implicated as targets in cancer [46]. We are currently investigating other disease models where PDE4 shortform activity underpin disease [7] and show in this study that KTX207 is much more potent than BI in preventing TNF release in immune cells (Fig. 6C).

In summation, we report on the discovery of a novel PDE4 shortform selective degrader and the evolution of the well‐characterized PDE4 inhibitor, BI 1015550 into a PROTAC with notable improvements in selectivity, anti‐inflammatory potency, and longevity of action. This report opens the way for the use of targeted protein degradation as an improved route to circumvent the lack of selectivity and the limiting side effect profiles of conventional small molecule PDE4 inhibitors.

## Materials and methods

### Molecular modeling studies

Modeling experiments were performed using Molecular Operating Environment (MOE) software (Chemical Computing Group, Montreal, Quebec, Canada). The structures of the PROTAC KTX207, the BI 1015550, and the cereblon warhead were created in ChemDraw and subsequently imported and energy minimized in MOE. For the initial docking studies, the warheads also contained the start of the linker section. For subsequent ternary structure prediction, only the warheads were used as templates.

A crystal structure of PDE4D in complex with apremilast (PDB 7CBQ) was prepared for docking using the MOE Quickprep tool before deleting the second subunit of this protein dimer, as well as additional waters, ions, and other small ligands. For the docking of the E3 ligase warhead to cereblon, a crystal structure consisting of DDB1 bound to cereblon and complexed with thalidomide (PDB 4CI1) was selected. The structure was prepared for docking in the same way as for PDE4D and DDB1 removed. All docking studies were performed using the MOE Docking tool. Initially, only the PDE4D and cereblon warheads alone were docked into the PDE4D and cereblon‐binding pockets using apremilast and thalidomide, respectively, as a template. The best‐docked pose was selected and then used as a template to dock the entire KTX207 compound into the binding pocket. For generating the ternary complex between PDE4D, cereblon, and KTX207, the structure of PDE4D and cereblon bound to their warheads only were used as templates. The ternary complex was modeled using the MOE three body search method4B interface and clustered using the ppd cluster 5 function. Docked poses were visually inspected to select the best pose.

### Cell culture, transfection, and compound treatment

HEK293 (ATCC, Cat #CRL‐1573, RRID: CVCL_0045) and human non‐small‐cell lung cancer cell lines A549 (ATCC, Cat #CCL‐185, RRID: CVCL_0023) were obtained from the American Type Culture Collection (LGC Standards, Teddington, Middlesex, UK). All cell lines were free from mycoplasma and other microbial contaminants. Cells were cultured in DMEM with 2 mm l‐glutamine supplemented with 10% FBS and 1% penicillin–streptomycin at 37 °C in a humidified atmosphere with 5% CO_2_. Cells were split at 80–90% confluence, and media was replaced every 2–3 days as required. Transfections of HEK293 cells were carried out using Lipofectamine 3000 (Life Technologies, Carlsbad, CA, USA) as per the manufacturer's instruction. DNA plasmid constructs used for transfection were pcDNA3‐PDE4D2‐VSV, pcDNA3‐PDE4D5‐VSV, pcDNA3‐QUAD‐PDE4D5‐VSV (K48R:K53R:K78R:K140R) mutant, and pcDNA3‐PDE4A7‐VSV. Mock transfection was done by addition of transfection reagent only without plasmid DNA. Bifunctional degrader KTX207 and warhead control BI 1015550 were provided by Katalytic Therapeutics (San Diego, CA, USA). Bortezomib was kindly provided by Dr Thimo Kurz (University of Glasgow). All chemicals were dissolved in DMSO (final concentration of 0.01%). Unless mentioned otherwise, cells were typically harvested 24 h after treatment.

### Western blotting

A549 cellular lysates were prepared in lysis buffer (25 mm Tris/HCl pH 7.4, 150 mm NaCl, 1% NP‐40, 1 mm EDTA, 5% glycerol) with complete EDTA‐free protease inhibitor cocktail tablets (Roche, West Sussex, UK). Protein concentration of lysates was determined using the Bradford assay, and all samples were equalized for protein concentration. Proteins were separated by SDS/PAGE (4–12% Bis‐Tris gels) and transferred onto nitrocellulose membranes for western blotting. The membranes were blocked in Intercept (TBS) Blocking Buffer (LI‐COR) for 1 h at room temperature, followed by incubation with primary antibody dilution in Intercept T20 (TBS) Antibody Diluent overnight (LI‐COR) at 4 °C with gentle shaking. Primary antibodies used were rabbit anti‐PDE4D (#ab171749, 1:3000; Abcam, Cambridge, UK), sheep anti‐PDE4D (1:5000; In‐house), sheep anti‐PDE4B (1:5000; In‐house), mouse anti‐GAPDH (# 60004‐1‐Ig, 1:80 000; Proteintech, Manchester, UK), rabbit anti‐GAPDH (#ab181602, 1:5000; Abcam), mouse anti‐ubiquitin (#sc‐8017, 1:1000; Santa Cruz), rabbit anti‐phospho‐(Ser/Thr) PKA substrate (#9621, 1:1000; Cell Signaling, Beverly, MA, USA), rabbit anti‐phospho‐ERK (#4377, 1:1000; Cell Signaling), mouse anti‐total ERK (#4696, 1:1000; Cell Signaling), rabbit anti‐PARP1 (#9542, 1:1000; Cell Signaling), mouse anti‐Ki67 (#sc‐23 900. 1:1000; Santa Cruz Biotechnology, Dallas, TX, USA), and rabbit anti‐VSV‐G (#ab1874, 1:3000; Abcam). Membranes were washed with TBST and then incubated with fluorescently labeled secondary antibodies (1:20 000) for 1 h at room temperature. The secondary antibodies used were Alexa Fluor 680 donkey anti‐goat IgG (#ab175776; Abcam), IRDye 680RD donkey anti‐mouse IgG (#926‐68 072), IRDye 800CW donkey anti‐mouse IgG (#926‐32 212), and IRDye 800CW donkey anti‐rabbit IgG (#926‐32 213). Images were acquired using Li‐Cor Odyssey CLx Imaging System, and signals were detected at 700 and 800 nm channels. All densitometry analyses were done using Image Studio (LI‐COR) and normalized to GAPDH. Representative images were shown in grayscale.

### Measurement of cAMP response

Stable A549 cell line expressing CRE‐Luc (pGL4.29[*luc2P*/CRE/Hygro]) vector (#E847A; Promega, Chilworth, Southampton, UK) was generated by transient transfection using Lipofectamine 3000 (Life Technologies) as per the manufacturer's instruction, followed by 200 μg·mL^−1^ hygromycin selection. The stable cells were seeded on Corning 96‐well white plate with flat clear bottom at 10000 cells·well^−1^ a day prior to assay. The growth medium was removed and replaced with 100 μL of phenol‐red free medium containing 1 nm KTX207 or BI and 100 μm d‐Luciferin sodium salt (#5427; Tocris Bioscience, Abingdon, Oxfordshire, UK) for 24 h. Ten micromolar of forskolin (FSK) was added in the last 3 h to induce the reporter gene expression. The luminescence response was measured before and after FSK was added to the cells using a Berthold Tristar 5 microplate reader.

### Immunostaining

A549 cells were plated onto sterile glass coverslips. After 24 h incubation on coverslips the cells were fixed using 4% (w/v) paraformaldehyde for 15 min at room temperature and permeabilized with 0.1% Triton X‐100 in PBS for 10 min. After three washes with PBS, the cells were blocked with 10% donkey serum and 2% BSA (w/v) in TBS for 2 h followed by three washes with TBS. The primary antibodies sheep anti‐PDE4D or rabbit anti‐PDE4D1 (1:4000; In‐house) were diluted to the required concentration in blocking buffer diluted 1:1 with PBS and incubated overnight at 4 °C. After three washes in PBS, cells were incubated with diluted secondary antibodies Alexa Fluor 594 donkey anti‐sheep IgG (Alexa Fluor #A11016, 1:500) or Alexa Fluor 488 donkey anti‐rabbit IgG (#A21206, 1:500) for 1 h. Cells were washed three times before being mounted to glass slides using ProLong Gold Antifade Mountant with DAPI (Invitrogen). Images were acquired on a Zeiss LSM 880 confocal laser scanning microscope controlled with the Zeiss ZEN imaging software, using a 63× Plan‐Apochromat 1.4NA DIC oil immersion objective. Images were then quantified using ImageJ Gel Analyzing tool (NIH, Bethesda, MA, USA).

### Cell infection and FRET microscopy

A549 cells were seeded onto 25 mm glass coverslips at a density of 15 000 cells per coverslip and incubated for 24 h. Following this, the cells were infected with adenoviral E1‐camps FRET‐based reporter at a concentration of 100 pfu·cell^−1^. To determine the optimal isoprenaline concentration (Sigma‐Aldrich, Gillingham, Dorset, UK), cells were washed with FRET buffer (composed of 144 mm NaCl, 5.4 mm KCl, 1 mm MgCl_2_, 2 mm CaCl_2_, and 10 mm HEPES, pH 7.4) after 48 h and treated with increasing concentrations of isoprenaline (ranging from 100 nm to 10 μm), followed by a saturator consisting of 10 μm FSK and 100 μm IBMX (Tocris, UK). For the PROTAC experiments, cells were incubated with either 1 nm or 10 nm of KTX207 or BI 1015550, or vehicle (DMSO) as a control. Data acquisition was performed using a multi‐cell FRET microscopy system, as previously described [47], and each subsequent pharmacological treatment was introduced after the establishment of a plateau.

### 3D spheroid formation and spheroid size evaluation

Two hundred microliters of complete medium containing 3750 of A549 cells were seeded in each well of Nuclon Sphera 96‐well round‐bottomed plate (VWR). Plates were centrifuged at 1500 rpm for 10 min to promote cell proximity. Treatments were performed by adding 50 μL of fresh complete medium with indicated compounds 72 h later. The spheroids were visualized on an inverted phase‐contrast microscope with 4x magnification (Nikon Eclipse Ts2), and images were obtained with the DS‐Fi3 camera and captured through NIS Elements (Nikon) on day 0 (before treatment start) and every 3–4 days after treatments. Spheroid size was evaluated by measuring spheroid perimeter and roundness using imagej software (NIH, Bethesda, MA, USA).

### T‐cell isolation and activation

Peripheral blood mononuclear cells (PBMCs) were isolated from healthy donors following written informed consent (approved by the Ethics Committee of the College of Medical, Veterinary and Life Sciences, University of Glasgow and The Scottish National Blood Transfusion Service (SNBTS Ref Number: SG2023‐02) via density gradient centrifugation using Lymphocyte Separation Medium (Corning, Inc., New York, NY, USA)). The study methodologies conformed to the standards set by the Declaration of Helsinki. Subsequently, PBMCs were seeded in culture dishes to facilitate the adhesion of undesirable monocytes in AIM‐V medium (Gibco, Waltham, MA, USA) supplemented with 5% human AB serum (Sigma‐Aldrich). T cells were activated for 72 h with Phytohemagglutinin‐L (PHA‐L) at a concentration of 5 μg·mL^−1^ (Roche, Basel, Switzerland), along with recombinant human interleukin (rhIL)‐2 (10 ng·mL^−1^), rhIL‐7 (5 ng·mL^−1^), and rhIL‐15 (20 mg·mL^−1^) (Thermo Fisher Scientific, MA, USA). PHA‐activated and nonactivated T cells were treated with 10 μm KTX207 or BI compound for 24 h. Cells were then lysed in RIPA lysis buffer and subjected to western blot.

### LPS‐induced TNF‐α PBMC assay

Normal PBMCs (iXCells # 10HU‐003; San Diego) were thawed, resuspended in RPMI supplemented with 10% FBS and 1% penicillin–streptomycin. One million cells·mL^−1^ were plated in 96‐well plates in 100 μL per well. Lipopolysaccharide (LPS) (100 ng·mL^−1^) and compounds in serial dilutions were then added to cells in 100 μL volume of complete media. Plates were incubated for 6 h at 37 °C/5% CO_2_, followed by centrifugation at 1200 rpm for 5 min. Media was decanted, and plates were washed with 200 μL of complete media. The plates were then added with 100 μL of fresh media. The wash out plates were added another 100 μL of media + LPS. The nonwash out plates were added with 100 μL of media + LPS + the serial dilutions of compounds. Plates were incubated for 18 h at 37 °C/5% CO_2_. The next day, plates were centrifuged at 1200 rpm for 5 min. Cell culture supernatant were collected and LPS‐induced TNF‐α production assay was performed using U‐Plex Human TNF‐α assay kit (Meso Scale Discovery, Rockville, Maryland) according to the manufacturer's instructions.

### Impedance‐based growth measurement using xCELLigence

Cell proliferation was monitored in real time using RTCA xCELLigence SP system (Roche), which was placed in an incubator at 37 °C with regulated 5% CO_2_. Fifty microliters of culture medium supplemented with 2% FBS was added to each well of E‐Plate 96 (Roche) for the impedance background measurement. An initial population of 4000 cells·well^−1^ of A549 and HEK293 in 100 μL of suspension in media were seeded in each well after background measurements were taken. Following cell adhesion, cells were treated with 1 nm of the bifunctional degrader molecules. After adding the cells and/or drugs, the final volume was 200 μL·well^−1^. The cultures were continuously monitored for up to 120 h, and the impedance as reflected by cell index (CI) values was set to record every 15 min. The xCELLigence data were then analyzed using the RTCA software (Roche). The results were expressed by normalized CI, which were derived by normalizing to the time of compound addition (T = 0 h). The rate of A549 and HEK293 cell growth was determined by measuring the slope of the line between the 25‐ and 65‐h and 10‐ and 35‐h intervals, respectively.

### Statistical analysis

All statistical analyses were performed using GraphPad Prism 8 software. Values are presented as mean ± SEM from at least three independent experiments. Statistical significances (compared to BI 1015550) were determined by a two‐tailed unpaired Student's *t*‐test. Values were considered significant if *P* < 0.05. Where representative immunoblots or microscopic images were shown, similar data were obtained *n* ≥ 3 times.

## Conflict of interest

CS, KC, and DS are founders of Katalytic Therapeutics and hold patent rights related to KTX207. The authors declare no conflict of interest.

## Author contributions

YYS devised and conducted most of the experiments, analyzed data, and wrote the manuscript. AG, PR, LGC, EG, ZT, TF, and EK performed research. AJ conducted FRET experiments and analyzed data. AZ undertook molecular modeling. JG devised FRET experiments and wrote the manuscript. PTY provided materials and devised T‐cell experiments. ET, CS, KC, and DS provided materials and wrote the manuscript. GSB devised experiments and wrote the manuscript. All authors read and approved the submitted version.

## Peer review

The peer review history for this article is available at https://www.webofscience.com/api/gateway/wos/peer‐review/10.1111/febs.17359.

## Data Availability

The data that support the findings of this study are openly available in Figshare at https://doi.org/10.6084/m9.figshare.27320418.v1.
